# Cerebral ischemia-reperfusion injury: mechanisms and promising therapies

**DOI:** 10.3389/fphar.2025.1613464

**Published:** 2025-07-16

**Authors:** Mingming Yang, Boya Liu, Bingqian Chen, Yuntian Shen, Guangliang Liu

**Affiliations:** ^1^ Department of Pediatrics, Binhai County People’s Hospital, Yancheng, Jiangsu, China; ^2^ Key Laboratory of Neuroregeneration of Jiangsu and Ministry of Education, Co-Innovation Center of Neuroregeneration, NMPA Key Laboratory for Research and Evaluation of Tissue Engineering Technology Products, Nantong University, Nantong, Jiangsu, China; ^3^ Department of Orthopaedics, Changshu Hospital Affiliated to Soochow University, First Peoples’ Hospital of Changshu City, Changshu, Jiangsu, China

**Keywords:** cerebral ischemia-reperfusion injury, traditional drugs, antioxidants, neuroprotection, exosomes, non-coding RNA

## Abstract

Cerebral ischemia-reperfusion injury (Cerebral I/R injury) is a critical pathological process following ischemic stroke, closely associated with multiple mechanisms including oxidative stress, neuroinflammation, and neuronal apoptosis. It also involves the alteration and regulation of numerous key genes and non-coding RNAs. Due to its complex regulatory mechanisms, there are currently no Food and Drug Administration (FDA)-approved drugs specifically targeting Cerebral I/R injury. Developing effective therapeutic strategies for Cerebral I/R injury remains a significant challenge in medical research. This review summarizes current treatment approaches for Cerebral I/R injury, which include traditional drugs, antioxidants, neuroprotective agents, exosomes, noncoding RNA therapeutics and combined intervention therapy. Pharmacotherapies exert positive effects on Cerebral I/R injury through antioxidative, anti-inflammatory, and neuroprotective mechanisms. Exosomes and noncoding RNA therapeutics can mitigate brain cell damage and promote neural function recovery by regulating the expression of downstream key genes via miRNAs, demonstrating potential as novel therapeutic options. Emerging evidence indicates that combined therapeutic strategies incorporating nanoparticle-mediated targeting demonstrate efficacy in treating cerebral I/R injury. By exploring the mechanisms of action and clinical application prospects of these different treatment strategies, this review aims to provide new insights and methods for the clinical management of Cerebral I/R injury.

## 1 Introduction

The brain is one of the most critical organs in the human body, requiring a substantial blood supply to provide oxygen and nutrients. Ischemic stroke, primarily caused by inadequate cerebral blood flow, results in loss of brain function and accounts for approximately 70%–80% of all stroke cases (Sakai and Shichita, 2019; Sommer, 2017). The primary clinical treatment strategy for ischemic stroke involves intravenous thrombolysis with tissue plasminogen activator (tPA) within 4.5 h or mechanical thrombectomy within 24 h to restore cerebral blood flow, a process known as reperfusion (Li M. et al., 2022). However, while reperfusion can alleviate the ischemic condition in brain tissue, it can also cause damage to neurons, glial cells, and other cell types, exacerbating the inflammatory response and brain injury. This phenomenon is referred to as Cerebral Ischemia-Reperfusion Injury (Cerebral I/R injury). The mechanisms underlying Cerebral I/R injury include excitotoxicity from excitatory amino acids, oxidative stress, calcium overload, mitochondrial dysfunction, endoplasmic reticulum stress, neuroinflammation, and disruption of the blood-brain barrier (BBB). The interplay of these mechanisms can ultimately lead to severe neuronal death and neurological dysfunction, further aggravating brain injury. Key regulatory molecules and signaling pathways, as well as non-coding RNAs such as microRNAs (miRNAs), long non-coding RNAs (lncRNAs), and circular RNAs (circRNAs), play crucial roles in the pathogenesis of Cerebral I/R injury.

The treatment strategies for Cerebral I/R injury remain a prominent and challenging focus in medical research. In recent years, numerous therapeutic modalities have been proposed and extensively investigated, including traditional drug therapies, antioxidants, neuroprotective agents, exosomes, noncoding RNA therapeutics and combined intervention therapy. These approaches aim to mitigate or reverse neuronal damage caused by the ischemia/reperfusion process and promote the recovery of neural function (Mao R. et al., 2022; Jurcau and Simion, 2021). Traditional drug therapies protect brain tissue through multiple mechanisms; antioxidants and neuroprotective agents exert relatively specific neuroprotective effects; exosome and noncoding RNA therapeutics represent highly promising emerging approach for repairing and reconstructing damaged tissues (Hamblin and Lee, 2021; Hermann et al., 2024; Winkle et al., 2021). While each of these treatment methods offers distinct advantages, they also face significant challenges and limitations. Promising preclinical findings demonstrate that nanoparticle-based targeting systems combined with adaptive interventions hold significant potential for addressing the dynamic pathological progression of cerebral I/R injury. This review integrates the latest research advancements to provide a comprehensive analysis of the treatment strategies for Cerebral I/R injury, focusing on traditional drug therapies, antioxidants, neuroprotective agents, exosome-based treatments, noncoding RNA therapeutics and combined intervention therapy with the goal of offering novel insights and approaches for the clinical management of Cerebral I/R injury.

## 2 Cerebral I/R injury mechanisms

### 2.1 Pathological process of cerebral I/R injury

**FIGURE 1:**
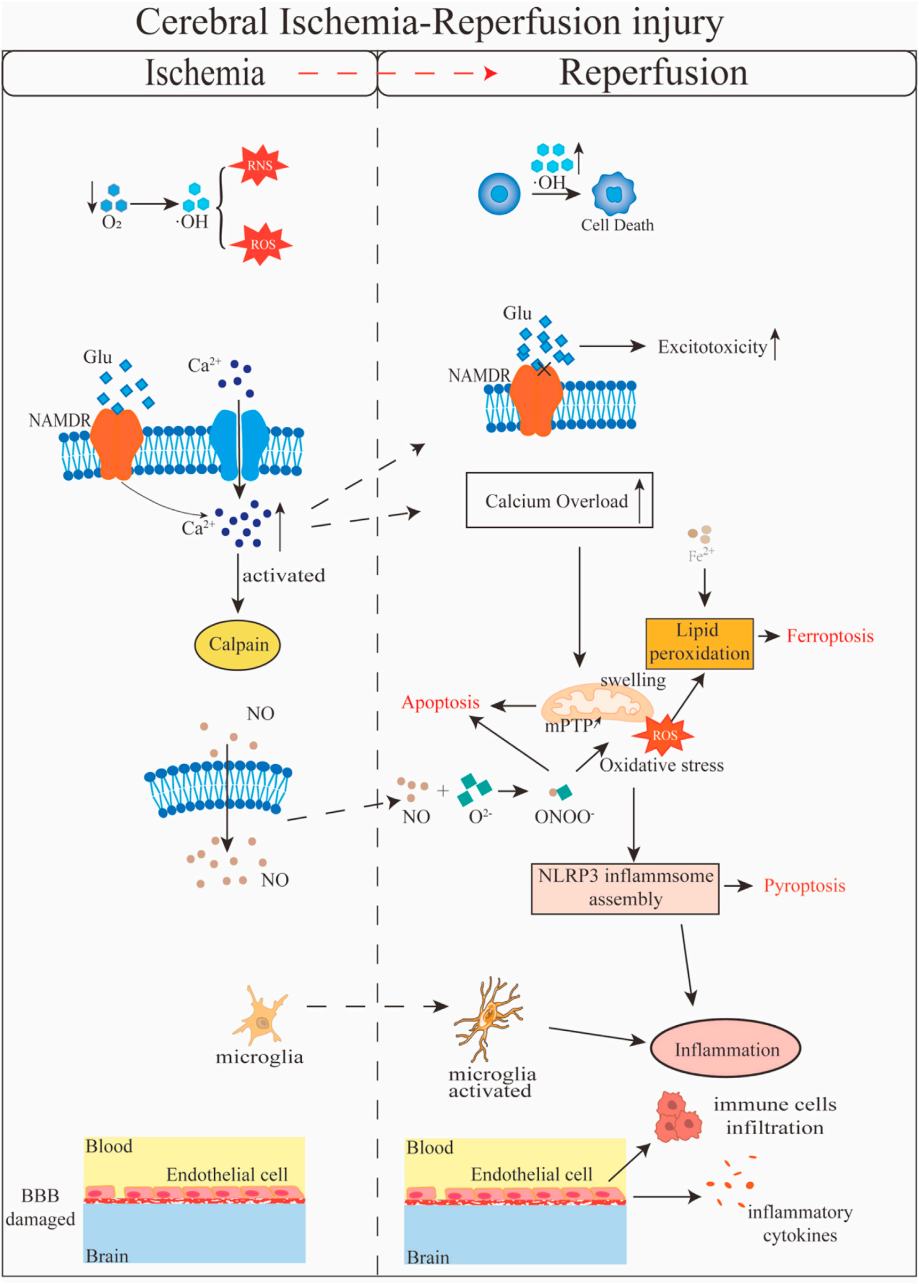
Schematic diagram of the molecular mechanisms involved in cerebral ischemia-reperfusion injury.

Cerebral I/R injury is a complex pathological process involving multiple mechanisms (Figure 1). Excitatory amino acids, such as glutamate, play a critical role in Cerebral I/R injury. During ischemia, the excessive release of glutamate from synaptic vesicles leads to an influx of calcium ions, triggering excitotoxicity and subsequent cell death. Upon reperfusion, the restoration of energy metabolism disrupts the balance between glutamate release and uptake, further exacerbating excitotoxicity (Yu et al., 2023). Simultaneously, nitric oxide (NO) production during Cerebral I/R injury may exceed physiological levels, leading to cellular toxicity. NO reacts with superoxide anions to form peroxynitrite, which induces oxidative stress and cellular damage (Wang Y. et al., 2022; Terpolilli et al., 2012; Bolanos and Almeida, 1999). Free radicals, including reactive oxygen species (ROS) and reactive nitrogen species (RNS), are closely associated with Cerebral I/R injury. During ischemia, insufficient oxygen supply results in the accumulation of free radicals within cells, causing protein dysfunction, DNA damage, and lipid peroxidation (Sun et al., 2018). Upon reperfusion, the generation of these free radicals increases further, reacting with cell membranes, proteins, and nucleic acids, thereby damaging cellular structure and function. Mitochondria, which produce approximately 90% of cellular ROS, are particularly vulnerable. Cerebral I/R injury enhances mitochondrial ROS production, leading to mitochondrial dysfunction (Kumar Saini and Singh, 2024). Calcium overload is another crucial mechanism in Cerebral I/R injury. During ischemia, intracellular calcium ion concentrations rise, activating calcium-dependent enzymes and disrupting the cytoskeleton. Upon reperfusion, calcium ions flood into cells, exacerbating calcium overload, increasing mitochondrial membrane permeability, and triggering both apoptosis and necrosis (Du et al., 2021; Yin et al., 2019). Additionally, mitochondrial biogenesis, dynamic changes, and abnormal autophagy all contribute to the exacerbation of brain I/R injury. In the early stages of reperfusion, autophagy plays a protective role by clearing damaged organelles and misfolded proteins. However, excessive autophagy in later stages can lead to cell death and secondary tissue damage (Huang et al., 2019). Similarly, endoplasmic reticulum stress (ERS) and the unfolded protein response (UPR) initially provide protection by promoting adaptive responses (Shen et al., 2021; Wang et al., 2020; Li R. et al., 2021). However, prolonged oxidative stress and calcium overload during reperfusion exacerbate misfolded protein accumulation and severe ERS, leading to UPR failure and activation of apoptotic pathways (Jiang et al., 2023; Yang M. et al., 2024). Furthermore, Cerebral I/R injury activates microglia within the central nervous system and induces infiltration and accumulation of peripheral immune cells (Wu et al., 2020a). Damage-associated molecular patterns (DAMPs) released from necrotic tissues trigger the activation of resident microglia, which in turn elevate inflammatory cytokines and promote the recruitment of neutrophils, monocytes, and lymphocytes to the central nervous system, thereby widely activating peripheral immune responses (Chen H. et al., 2020). Concurrently, Cerebral I/R injury damages cerebral microvascular endothelial cells and compromises BBB. Post-injury, endothelial cell permeability increases, leading to BBB disruption, which facilitates further infiltration of peripheral immune cells, exacerbates inflammation, and contributes to more severe tissue damage (Henrot et al., 2023; Shi et al., 2020).

Cell death is a critical mechanism underlying I/R injury and serves as an important pathological indicator. In neuronal cells, the restoration of cerebral blood flow can trigger multiple forms of cell death, including apoptosis, necroptosis, pyroptosis, panoptosis, and ferroptosis (Tuo et al., 2022a). During ischemia, mitochondrial dysfunction leads to increased membrane permeability, resulting in the release of cytochrome c and activation of the Caspase signaling cascade (Datta et al., 2020). In Cerebral I/R injury, necroptosis regulated by the RIPK1/RIPK3/MLKL signaling pathway plays a pivotal role (Liao et al., 2020; Li J. et al., 2019; Wang et al., 2018). Pyroptosis is prevalent in the early stages of ischemia-reperfusion and is closely linked to inflammatory responses. The Nucleotide-binding oligomerization domain, leucine-rich repeat and pyrin domain-containing 3 (NLRP3) inflammasome is a key mediator in this process, and several drugs and inhibitors have been shown to reduce neuronal damage and cerebral infarction following Cerebral I/R injury by inhibiting NLRP3-dependent pyroptosis (Wang L. et al., 2022; Kang et al., 2021; Xiao et al., 2021). Recently, researchers have identified a unique form of inflammatory programmed cell death during Cerebral I/R injury, termed PANoptosis (Yan et al., 2022; Lan et al., 2024). Z-DNA binding protein 1 (ZBP1) and transforming growth factor-β-activated kinase 1 (TAK1) are central regulators of PANoptosis, although the precise mechanisms remain to be fully elucidated (Qiu et al., 2024). Ferroptosis, characterized by lipid peroxidation and iron overload, is also a significant contributor to neuronal death in Cerebral I/R injury (Tuo et al., 2022b). During cerebral ischemia-reperfusion, disruption of the BBB leads to iron dyshomeostasis, while excessive ROS generation further promotes ferroptosis (She et al., 2023). These various cell death pathways act synergistically in Cerebral I/R injury, and ongoing research may identify these signaling pathways as potential therapeutic targets.

### 2.2 Key molecules and potential therapeutic targets for cerebral I/R injury

#### 2.2.1 Regulatory molecules and signaling pathways

With our in-depth understanding of the pathological process of cerebral ischemia-reperfusion injury, an increasing number of related molecules involved in the regulation of pathways such as oxidative stress, inflammatory response, and apoptotic signaling have been found to play a crucial role in the occurrence of cerebral ischemia-reperfusion injury. The chemokine (C-C motif) ligand 2/chemokine receptor 2 (CCL2/CCR2) signaling pathway is implicated in acute neuroinflammation and BBB dysfunction following cerebral ischemia-reperfusion. Clinical data indicate that elevated levels of CCL2 in blood or cerebrospinal fluid correlate with clinical symptoms in stroke patients and serve as a prognostic biomarker (Geng et al., 2022; Xu Q. et al., 2024; Liu S. et al., 2023). In recanalized stroke patients, circulating Caveolin-1 (Cav-1) levels are reduced; however, enhancing Cav-1 expression in endothelial cells in middle cerebral artery occlusion/reperfusion (MCAO/R) injury mice significantly reduces infarct volume, decreases vascular permeability, and mitigates thrombo-inflammation (Zhang X. et al., 2022). Platelet-specific deletion of Atypical Chemokine Receptor 3 (ACKR3) enhances platelet activation and thrombosis, exacerbating inflammation and brain tissue damage (Rohlfing et al., 2022). Tripartite Motif Containing 45 (TRIM45) is highly expressed in the peri-infarct region of MCAO/R-treated mice and plays a pivotal role in neuroinflammation via the NF-κB signaling pathway (Xia et al., 2022). Recent studies show that Cav3.2 knockout attenuates oxidative stress, inflammatory response, and neuronal apoptosis through the calcineurin/NFAT3 pathway (Dai et al., 2024). The transient receptor potential cation channel subfamily C member 6 (TRPC6) activation prevents neuronal death, while its blockade increases ischemic sensitivity (Li W. et al., 2019; Liu et al., 2020a; Shekhar et al., 2021; Liu et al., 2020b). Additionally, Rnf213 upregulation correlates with neuronal apoptosis and infarct volume, and RACK1 knockdown exacerbates apoptosis (Li et al., 2023a; Zhao et al., 2024). Furthermore, the latest research has found that some deubiquitinating enzymes (DUBs) play a dual role in cerebral ischemia-reperfusion injury, influencing the extent of injury by regulating processes such as inflammation, oxidative stress, and cell death (Qin X. et al., 2025). In conclusion, as we gain deeper insights into the molecular mechanisms of cerebral ischemia-reperfusion injury, more precise and effective therapeutic strategies can be developed to mitigate brain damage and improve patient outcomes. Future research will continue to explore the interactions among these molecular mechanisms and their specific roles in cerebral ischemia-reperfusion injury, offering new hope for stroke patients.

#### 2.2.2 Non-coding RNAs

Non-coding RNAs, including miRNAs, lncRNAs, and circRNAs, play essential roles in regulating cerebral I/R injury through the modulation of neuroinflammation, oxidative stress, and apoptosis (Table 1). These ncRNA classes synergistically govern BBB integrity and intercellular crosstalk emerging as pivotal therapeutic targets in cerebrovascular disorders. The expression profile of miRNAs in cerebral I/R injury undergoes dynamic alterations, critically contributing to neuroinflammation, oxidative stress, and neuronal apoptosis (Maitrias et al., 2017; Xu et al., 2025; Neag et al., 2022). miR-19a/b-3p exacerbates neuroinflammation by activating the SIRT1/FoxO3/SPHK1 pathway, amplifying pro-inflammatory cytokine production, while miR-328-3p enhances TNF-α and IL-6 expression, promoting neutrophil infiltration (Zhou et al., 2021; Wang S. et al., 2021). miR-670 induces phosphorylation-mediated degradation of Yes-associated protein (YAP), exacerbating neuronal apoptosis and neurological deficits, whereas miR-30a-5p disrupts BBB integrity through ZnT4/zinc dyshomeostasis (Yu et al., 2021; Wang P. et al., 2021). Conversely, miR-532-5p suppresses the CXCL1/CXCR2/NF-κB axis, and miR-652 mitigates oxidative stress by targeting NOX2 (Shi et al., 2021; Zuo et al., 2020). Emerging regulators include miR-124, which inhibits STAT3 phosphorylation to attenuate microglial activation and pyroptosis, and miR-139-5p, activating the Nrf2 antioxidant pathway to counteract ROS/TXNIP-driven NLRP3 activation (Sun et al., 2020; Yao et al., 2022). miR-223 directly binds NLRP3 mRNA to limit cytokine release, while astrocyte-derived exosomal miR-29a-3p suppresses TP53INP1/NF-κB signaling in microglia (Sha et al., 2019; Liu X. et al., 2022). Age-dependent duality is observed in miR-155-5p, where knockdown inhibits DUSP14/NF-κB signaling in young models but requires preservation in aged brains to maintain cognitive stability (Shi Y. et al., 2022; Que et al., 2020). Additionally, macrophage-derived exosomal miR-Novel-3 exacerbates ferroptosis and neuroinflammation by activating microglia (Qin et al., 2024). Collectively, miRNAs orchestrate I/R injury progression through synergistic modulation of inflammatory pathways, redox imbalance, BBB breakdown, and intercellular crosstalk, highlighting their pivotal roles in ischemic pathogenesis.

**TABLE 1:** Noncoding RNAs in cerebral ischemia-reperfusion injury.

Category	Name	Function & mechanism	References
microRNAs (miRNAs)	miR-19a/b-3p	Activates SIRT1/FoxO3/SPHK1 pathway to amplify pro-inflammatory cytokine production	Zhou et al. (2021)
	miR-328-3p	Enhances TNF-α and IL-6 expression, promoting neutrophil infiltration	Wang et al. (2021a)
	miR-670	Induces phosphorylation-mediated YAP degradation, exacerbating neuronal apoptosis	Yu et al. (2021)
	miR-30a-5p	Disrupts BBB integrity via ZnT4/zinc dyshomeostasis	Wang et al. (2021b)
	miR-532-5p	Suppresses CXCL1/CXCR2/NF-κB axis to attenuate neuroinflammation	Shi et al. (2021)
	miR-652	Reduces oxidative stress by targeting NOX2	Zuo et al. (2020)
	miR-124	Inhibits STAT3 phosphorylation to reduce microglial activation and pyroptosis	Sun et al. (2020)
	miR-139-5p	Activates Nrf2 antioxidant pathway to counteract ROS/TXNIP-driven NLRP3 activation	Yao et al. (2022)
	miR-223	Directly binds NLRP3 to limit inflammasome activity and cytokine release	Sha et al. (2019)
	miR-29a-3p	Suppresses TP53INP1/NF-κB signaling in microglia	Liu et al. (2022a)
	miR-155-5p	Exhibits age-dependent duality: inhibits DUSP14/NF-κB in young models but preserves cognitive function in aged brains	Shi et al. (2022a), Que et al. (2020)
	miR-Novel-3	Activates microglial ferroptosis and neuroinflammation	Qin et al. (2024)
lncRNAs	MALAT1	Interacts with STAT1 to enhance NLRP3 transcription, promoting microglial pyroptosis	Khoshnam et al. (2024), Tan et al. (2021), Zhao et al. (2023a), Fathy et al. (2021)
	MEG3	Competes with miR-485 to upregulate AIM2 inflammasome; aggravates mitochondrial dysfunction and oxidative stress	Li et al. (2022b), Yan et al. (2024), Yao et al. (2024)
	KCNQ1OT1	Sponges miR-153-3p/miR-140-3p/miR-30b/miR-9 to activate Foxo3-mediated pyroptosis; inhibits autophagy via miR-200a/FOXO3/ATG7	Yu et al. (2019), Yi et al. (2020), Li et al. (2020a)
	XIST	Sponges miR-96-5p to activate NLRP3 inflammasome and IL-1β secretion	Zhang et al. (2021)
	TUG1	Disrupts BBB integrity through miR-145/AQP4 dysregulation in astrocytes	Shan et al. (2020), Yin et al. (2021)
	LOC102555978	Directly activates NLRP3-dependent microglial pyroptosis	Gao et al. (2024)
	FENDRR	Amplifies NLRC4 inflammasome signaling in diabetic CIRI models	Wang et al. (2021c)
circRNAs	circTLK1	Binds miR-335-3p to promote TCDD-inducible PARP overexpression, worsening neuronal damage	Wu et al. (2019)
	circ-HECTD1	Regulates astrocyte activation (miR-142/TIPARP axis) and neuronal apoptosis (miR-133b/TRAF3 axis)	Han et al. (2018), Dai et al. (2021)
	circRIMS	Sponges miR-96-5p to activate JAK/STAT1 signaling, amplifying NLRP3 inflammasome activity	Li et al. (2023b)
	circCCDC6	Suppresses miR-128-3p to activate TXNIP/NLRP3 pathway	Wang et al. (2023a)
	circFOXP1	Inhibits STAT3/apoptotic signaling to mitigate brain injury	Yang et al. (2023a)
	circ-FoxO3	Stabilizes BBB integrity via mTORC1 activity	Yang et al. (2022a)
	circPTP4A2	Drives pro-inflammatory microglial polarization through STAT3 regulation	Wang et al. (2024a)

lncRNAs, non-coding transcripts exceeding 200 nucleotides, regulate ischemic injury through interactions with RNA, DNA, proteins, or RNA-binding proteins. A critical mechanism involves their role as molecular sponges for miRNAs, dynamically modulating gene expression under ischemic stress. Metastasis-associated lung adenocarcinoma transcript 1 (MALAT1), an evolutionarily conserved lncRNA, is highly expressed in ischemic stroke patients and exacerbates neuroinflammation by interacting with STAT1 to enhance NLRP3 transcription, driving microglial pyroptosis in diabetic models (Khoshnam et al., 2024; Tan et al., 2021; Zhao N. et al., 2023). This interaction amplifies NLRP3-mediated inflammatory cascades, linking MALAT1 dysregulation to poor clinical outcomes (Fathy et al., 2021). Maternally expressed gene 3 (MEG3), upregulated post-ischemia, activates pyroptosis via competitive binding to miR-485, which elevates AIM2 inflammasome components, while simultaneously exacerbating mitochondrial dysfunction and oxidative stress (Li T. H. et al., 2022; Yan et al., 2024; Yao et al., 2024). Potassium voltage-gated channel subfamily Q member one opposite strand 1 (KCNQ1OT1), significantly overexpressed in ischemic stroke, functions as a multi-miRNA sponge (miR-153-3p, miR-140-3p, miR-30b, miR-9) to promote Foxo3-mediated pyroptosis and MMP8-driven neuronal injury. Its knockdown mitigates autophagy via the miR-200a/FOXO3/ATG7 axis, reducing infarct size and endoplasmic reticulum stress (Yu et al., 2019; Yi et al., 2020; Li Y. et al., 2020). X-inactive specific transcript (XIST) exacerbates endothelial pyroptosis by sponging miR-96-5p, leading to NLRP3 inflammasome activation and IL-1β hypersecretion (Zhang et al., 2021). Other lncRNAs, such as taurine upregulated gene 1 (TUG1), disrupt BBB integrity through miR-145/AQP4 dysregulation in astrocytes, while LOC102555978 directly activates NLRP3-dependent microglial pyroptosis, and fetal-lethal noncoding developmental regulatory RNA (FENDRR) amplifies NLRC4 inflammasome signaling to intensify diabetic CIRI (Shan et al., 2020; Yin et al., 2021; Wang L. Q. et al., 2021; Gao et al., 2024). Additional lncRNAs like five prime to XIST (FTX), small nucleolar RNA host gene 12/14/15 (SNHG12/14/15), SOX2 overlapping transcript (SOX2OT), and CCAAT enhancer binding protein alpha antisense RNA 1 (CEBPA-AS1) further modulate ischemic injury via miRNA interactions, influencing oxidative stress, angiogenesis, and neuroinflammatory pathways (Wang J. et al., 2021; Wang et al., 2022c; Wang and Hu, 2021; Tu et al., 2022; Qi et al., 2017; Barangi et al., 2023). Collectively, lncRNAs orchestrate CIRI progression through inflammasome activation, pyroptosis amplification, redox imbalance, and multi-target miRNA sponging, positioning them as pivotal regulators of ischemic pathogenesis.

Circular RNAs (circRNAs), a class of covalently closed non-coding RNAs with enhanced structural stability, are emerging as pivotal regulators in CIRI, where they modulate neuronal injury and repair through miRNA-dependent mechanisms. These molecules, abundant in the nervous system, not only participate in physiological processes like neuronal development and synaptic plasticity but also drive pathological cascades in CIRI (Yang et al., 2018). For instance, circular RNA TLK1 (circTLK1) is upregulated in MCAO models and ischemic stroke patients, where it exacerbates neuronal damage by competitively binding miR-335-3p and promoting TCDD-inducible PARP overexpression (Wu et al., 2019). Similarly, circular RNA HECTD1 (circ-HECTD1) contributes to astrocyte activation and neuronal loss via the miR-142/TIPARP and miR-133b/TRAF3 axes, respectively (Han et al., 2018; Dai et al., 2021). Recent studies further reveal the involvement of circRNAs in inflammatory cell death pathways: circular RNA derived from RIMS1 (circRIMS), previously linked to cancer progression, amplifies NLRP3 inflammasome activity and inflammatory cytokine release by sponging miR-96-5p to activate JAK/STAT1 signaling in CIRI models, while circular RNA from coiled-coil domain-containing 6 (circCCDC6) enhances neuronal injury by suppressing miR-128-3p, leading to TXNIP/NLRP3 pathway activation (Li W. et al., 2023; Wang C. et al., 2023). Beyond these mechanisms, circular RNA ASXL2 (circASXL2), circular RNA CAMK4 (circCAMK4), and circular RNA UCK2 (circUCK2) exacerbate neuronal degeneration through miRNA dysregulation, whereas circular RNA FOXP1 (circFOXP1) and circular RNA FoxO3 (circ-FoxO3) counteract injury by inhibiting STAT3-mediated apoptosis and stabilizing BBB integrity via mTORC1 modulation (Zhang et al., 2024a; Zhang et al., 2020; Tang et al., 2020; Chen W. et al., 2020; Dai et al., 2022; Yang B. et al., 2021; Yang J. et al., 2023; Yang Z. et al., 2022). Notably, circular RNA PTP4A2 (circPTP4A2) drives microglial polarization toward a pro-inflammatory phenotype, aggravating neuroinflammation (Wang X. et al., 2024). The ongoing clinical trial NCT04175691 aims to decode circRNA-miRNA-lncRNA networks in stroke patients, highlighting their potential as diagnostic markers and therapeutic targets. Collectively, circRNAs act as molecular hubs in CIRI, bridging miRNA interactions, inflammatory cascades, and vascular damage, thereby offering multifaceted strategies for neuroprotection.

## 3 Therapeutic strategies for cerebral I/R injury

**FIGURE 2:**
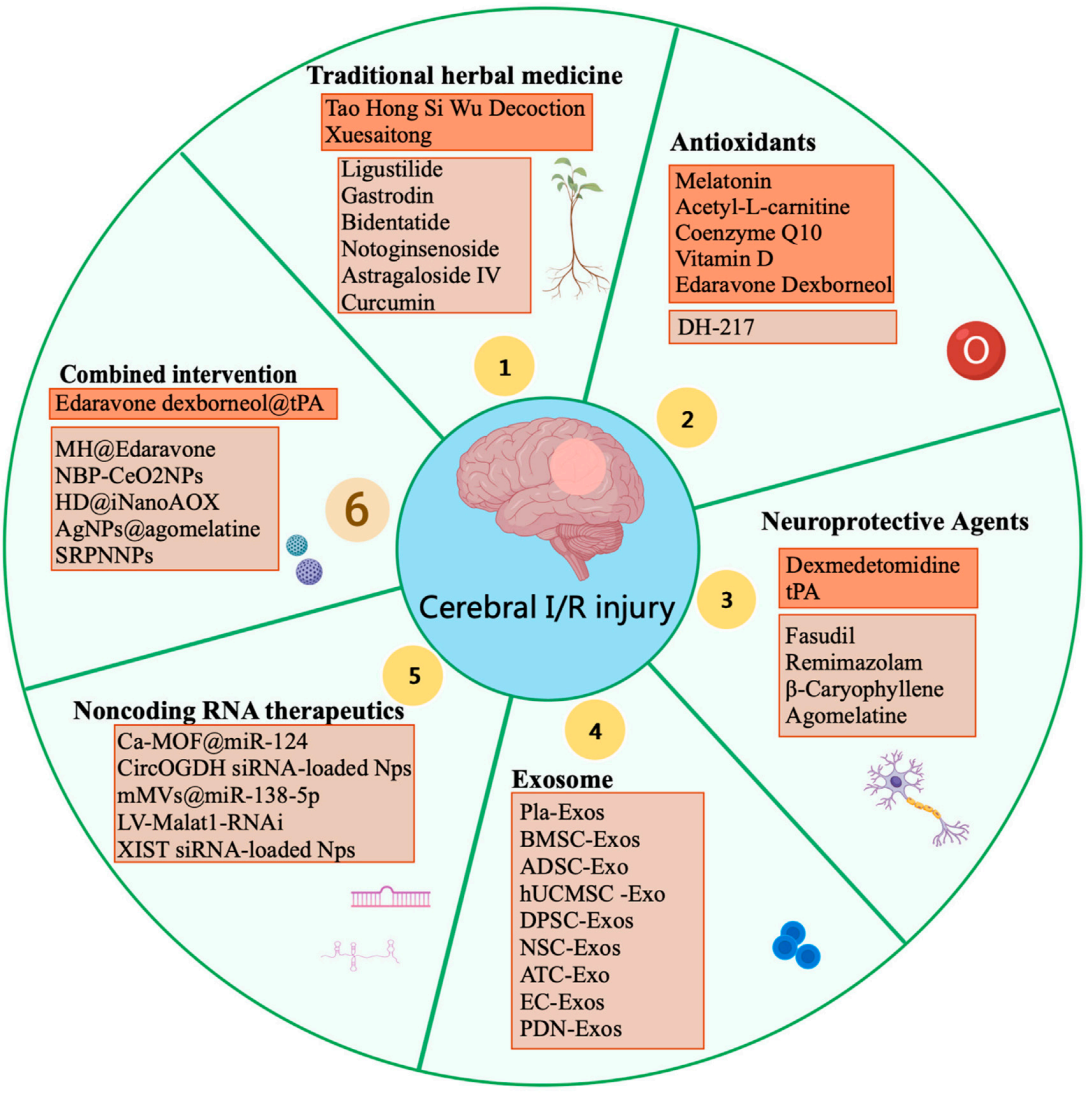
Therapeutic strategies for cerebral ischemia-reperfusion injury. Dark-colored boxes indicate clinically validated treatments, while light-colored boxes represent preclinical studies in animal models.

Cerebral I/R injury is a serious complication of ischemic stroke. Currently, there are no FDA-approved drugs specifically targeting brain I/R injury, underscoring the urgent need to develop more effective treatments. In recent years, advancements in drug research and development have led to the emergence of new drugs with high efficiency, low toxicity, and high bioavailability, which have shown promising therapeutic effects in treating Cerebral I/R injury (Figure 2). Therapeutic interventions for cerebral I/R injury are time-sensitive and stratified across distinct pathophysiological phases. In the hyperacute window (0–6h), rapid reperfusion with intravenous thrombolysis synergizes with dual-targeted antioxidant therapies to mitigate early oxidative damage. By 6–24h, interventions shift toward neuroinflammation modulation (Xu X. et al., 2024). Subsequently, delayed interventions post-reperfusion focus on modulating chronic inflammation, apoptosis, and neurovascular remodeling (Zhang H. et al., 2022). Active components derived from plants can mitigate the pathological processes induced by ischemia and reperfusion through various signaling pathways. Antioxidants and neuroprotective agents alleviate oxidative stress and promote the release of neurotrophic factors, thereby exerting neuroprotective effects. Exosome and noncoding RNA therapeutics have emerged as a promising research focus due to the therapeutic potential. Currently, the treatment of cerebral I/R injury is transitioning from single-agent therapies to a multi-faceted approach that integrates multiple mechanisms and pathways, aiming to achieve more effective and precise therapeutic outcomes.

### 3.1 Traditional herbal medicine

For thousands of years, herbal medicine has played a significant role in human health. Many herbs have been found to exert protective effects against cerebral I/R injury (Pang et al., 2025; Zhang et al., 2024b). The Tao Hong Si Wu Decoction (THSWD), composed of six herbs—peach kernel, safflower, rehmannia, angelica, peony, and ligusticum—is a widely used traditional Chinese medicine formula known for its functions in promoting blood circulation and regulating immunity. Studies have demonstrated that THSWD can promote angiogenesis, regulate mitochondrial autophagy, reduce ROS production, inhibit NLRP3-mediated inflammatory responses, and decrease neuronal apoptosis, thereby exerting neuroprotective effects in cerebral I/R injury (Chen F. F. et al., 2020; Ji et al., 2022). The main active component of angelica, ligustilide (LIG), has been shown to improve mitochondrial function by activating the AMPK signaling pathway and alleviate cerebral I/R injury through regulation of PINK1/Parkin-mediated mitochondrial autophagy (Wu et al., 2022; Mao Z. et al., 2022). Gastrodin demonstrates multi-mechanistic therapeutic efficacy against cerebral ischemia-reperfusion injury through antioxidative, anti-inflammatory, neuroprotective, and blood-brain barrier stabilizing effects, emerging as an effective natural therapeutic agent for ischemic stroke management (Qin W. et al., 2025). Our research team systematically characterized the neuroprotective profile of Achyranthes bidentata polypeptides, demonstrating their potent anti-apoptotic, anti-inflammatory, and microvascular-protective effects (Cheng et al., 2019). Through bioactivity-guided fractionation combined with HPLC-MS structural elucidation, we identified Bidentatide as the principal bioactive entity (Ding et al., 2021; Ge et al., 2021). Notoginsenoside, derived from the medicinal plant Panax notoginseng, primarily consists of ginsenosides Rb1, Rd, Rg1, Re, and notoginsenoside R1. Xuesaitong soft capsules, formulated from notoginsenoside, have been approved for clinical use in treating ischemic stroke. A recent randomized clinical trial involving 1,200 patients with acute ischemic stroke demonstrated that Xuesaitong significantly improves neurological function at 3 months post-treatment. Specifically, the proportion of patients achieving functional independence was significantly higher in the Xuesaitong group; furthermore, the average reduction in National Institutes of Health Stroke Scale (NIHSS) score was also significantly greater in the Xuesaitong group (Wu et al., 2023). In addition to notoginsenoside and ginsenoside Rg1, astragaloside IV has also shown great potential in treating cerebral I/R injury (Xiao et al., 2021; Wang L. et al., 2023; Xiao et al., 2022; Ling et al., 2024; Xi et al., 2024). It possesses antioxidant, anti-neuroinflammatory, autophagy-regulating, anti-apoptotic, anti-ferroptotic, BBB-protective, neuroregenerative, and angiogenic properties, providing therapeutic benefits during both the early and late stages of cerebral I/R injury (Zeng et al., 2022). A traditional Chinese medicine compound, Qilong Capsule, which contains multiple components such as astragaloside IV, calycosin-7-glucoside, and lumbrokinase, was found in a recent clinical study to significantly increase the proportion of patients achieving functional independence (modified Rankin Scale ≤2) at 12 weeks to 68.5% in the treatment group (Lyu et al., 2024). Curcumin, extracted from the rhizomes of Zingiberaceae plants, acts as a natural iron chelator, increasing GPX4 expression to inhibit ferroptosis, while also regulating autophagy and pyroptosis, thereby improving cerebral I/R injury (Zhang L. et al., 2023; Li F. et al., 2021). Both ginkgetin and muscone converge on PPAR-γ signaling to drive robust microglial M2 polarization, synergistically amplifying neuroprotective effects and functional rehabilitation after cerebral ischemia (Han et al., 2022; Liu F. et al., 2023; Tang et al., 2022). Furthermore, other compounds such as ginkgolide, rhein, quercetin, gomisin N, parthenolide, resveratrol, and formononetin, as well as the herbal formulation Shuanglu Tongnao, have also been found to have protective effects on cerebral I/R injury (Yang Y. et al., 2024; Li L. et al., 2023; Yu et al., 2022; Li R. et al., 2023; Liu H. et al., 2023; Ding et al., 2022; Zhai et al., 2025). These findings suggest that natural plant compounds or their combinations hold significant potential for the treatment of cerebral I/R injury, warranting further clinical investigation.

### 3.2 Antioxidants

Given the critical role of oxidative stress in the pathogenesis of cerebral I/R injury, antioxidant drugs such as melatonin, acetyl-L-carnitine, vitamin D,and coenzyme Q10 have been evaluated in clinical trials to assess their efficacy in improving neurological function in patients with cerebral ischemia over 1–3 months, demonstrating certain benefits (Brion et al., 2024). Edaravone, a widely used neuroprotective agent in clinical practice, is employed to improve neurological symptoms caused by acute cerebral infarction (Kobayashi et al., 2019; Xu et al., 2021). Recently, Edaravone dexborneol, a novel neuroprotective agent combining edaravone and borneol, has shown excellent efficacy in phase III clinical trials, significantly improving neurological function in patients with acute cerebral ischemia. To be specific, the pivotal trial demonstrated a significantly higher rate of functional independence at 90 days and greater improvement in NIHSS scores. Furthermore, subgroup analysis revealed a 35% increase in neurological recovery rates with sublingual administration (Xu et al., 2021; Fu et al., 2024). Edaravone dexborneol exhibits synergistic anti-inflammatory and antioxidative effects, inhibiting ferroptosis via activation of the Nrf-2/HO-1/GPX4 signaling pathway, regulating extracellular matrix integrin and PDGFB/PDGFR-β signaling, protecting against BBB damage induced by cerebral I/R, and suppressing NF-κB nuclear translocation to block AIM2 inflammasome assembly—thereby suppressing microglial/astrocytic hyperactivation and leukocyte infiltration (Xiao et al., 2024; Sun et al., 2024; Wang D. et al., 2024; Zhang et al., 2025). Recent study has also found that peroxiredoxin-1, an antioxidant enzyme, controls stroke-related microglial responses to mitigate acute ischemic stroke (Kim et al., 2022). Additionally, the novel dual-acting antioxidant DH-217—which exerts neuroprotective effects by modulating the IKKβ/Nrf2/HO-1 signaling pathway to mitigate oxidative stress injury—has demonstrated promising therapeutic benefits in animal models of MCAO/R (Shen et al., 2023). However, the challenge of effectively penetrating the BBB and delivering antioxidants to the brain remains a significant obstacle to translating these findings into clinical applications (Brion et al., 2024). The rapid development of nanotechnology offers new possibilities for overcoming this bottleneck. Nanodrugs that can penetrate the blood-brain barrier and efficiently clear ROS hold promise for addressing the limitations of current drugs for ischemic stroke. Recent studies have shown that various functional nanoparticles capable of ROS removal have exhibited positive effects in Cerebral I/R injury (Yuan et al., 2021; Chen et al., 2024; Hou and Brenner, 2024). For instance, cerium oxide nanoparticles loaded with Dl-3-n-butylphthalide (NBP-CeO_2_NPs) can maintain BBB integrity, inhibit neuroinflammation and neuronal apoptosis, thereby reducing cerebral infarction and brain edema, providing a potential new treatment for ischemic stroke (Li X. et al., 2022). Moreover, a novel pH-sensitive hirudin-loaded antioxidant nitroxide radical nanoparticle (HD@iNano^AOX^) has been shown to effectively improve ischemic brain injury in mice (Mei et al., 2025). Nano delivery of antioxidants has emerged as a promising treatment approach for Cerebral I/R injury, although further clinical validation is required.

### 3.3 Neuroprotective agents

Some neuroprotective agents can reduce the volume of cerebral infarction after ischemia without causing hemorrhage, alleviate cell damage post-ischemia, and protect the brain from reperfusion injury. Dexmedetomidine (DEX), an α2-adrenergic agonist with sedative properties, exhibits neuroprotective effects by reducing inflammatory responses and oxidative stress, inhibiting apoptosis, protecting the BBB, maintaining coagulation-anticoagulation balance, and preventing vasospasm (Hu Y. et al., 2022). A clinical study demonstrated that dexmedetomidine combined with targeted hemodynamic therapy can reduce neuroinflammation during craniocerebral surgery without adverse effects on hemodynamics (Chen P. H. et al., 2021). Recent studies have also shown that DEX can regulate multiple miRNAs, including miR-324-3p, miR-199a, and miR-205-5p, thereby exerting a protective effect on cerebral I/R injury (Burlacu et al., 2022; Chen Y. et al., 2021; Yang J. J. et al., 2021). The thrombolytic drug tPA exerts neuroprotective effects after reperfusion by increasing AMPK phosphorylation and FUNDC1 expression, thereby improving mitochondrial function in neurons and inhibiting apoptosis (Cai et al., 2021). The novel benzodiazepine agonist remimazolam and the Rho kinase inhibitor fasudil, which target the γ-aminobutyric acid A (GABAa) receptor, can reduce inflammatory responses by inhibiting the NLRP3 and TLR4/NF-κB pathways, promote the secretion of neurotrophic factors, and alleviate cerebral ischemia-reperfusion injury (Shi M. et al., 2022; Guo et al., 2023). β-Caryophyllene, a natural bicyclic sesquiterpene found in essential oils, has significant neuroprotective effects in reducing ischemic stroke damage through the regulation of ferroptosis and activation of the Nrf2/HO1 axis (Hu Q. et al., 2022). Agomelatine is a potential candidate for treating ischemic stroke by protecting the brain from cerebral I/R injury via inhibition of apoptosis (Chumboatong et al., 2017). Citrate-coated silver nanoparticles (AgNPs) loaded with agomelatine can further enhance the formation of the BBB by inhibiting oxidative stress and endoplasmic reticulum stress, providing neuronal protection in acute cerebral ischemia/reperfusion in rats (Gelen et al., 2024). Nano delivery of neuroprotective agents represents a cutting-edge approach to treating cerebral ischemia-reperfusion injury. By overcoming the limitations of conventional therapies and targeting multiple aspects of the injury process, this strategy holds significant promise for improving patient outcomes in the future.

### 3.4 Exosome

Exosomes contain a variety of bioactive molecules and can transmit complex intercellular signals (Wang et al., 2022d; Wang W. et al., 2021; Miron et al., 2000). Studies have demonstrated that exosomes promote the recovery from cerebral I/R injury by modulating immune responses, cell metabolism, and neuronal plasticity (Table 2) (Hermann et al., 2024; Xu K. et al., 2024). Circulating plasma exosomes, derived from multiple cell sources, are enriched in Heat shock 70 (HSP70) and can regulate ROS, inhibit mitochondrial-mediated neuronal apoptosis, and alleviate blood-brain barrier damage by activating tight junction proteins, thereby improving neurological function in MCAO/R mouse models (Jiang et al., 2020; Kang et al., 2019). Bone marrow mesenchymal stem cell-derived exosomes (BMSC-Exos) restore lysosomal function via the mTOR/TFEB pathway and mitigate cerebral I/R injury by targeting DAPK2 with miR-133a-3p (Liu H. et al., 2024; Yang X. et al., 2023). Exosomes from C-X-C chemokine receptor type 4 (CXCR4)-overexpressing BMSCs enhance angiogenesis, protect neurons, and improve acute stroke outcomes (Li X. et al., 2020). Moreover, BMSC-Exos induce microglial polarization from M1 to M2, alleviating neuronal pyroptosis and reducing cerebral I/R injury (Liu et al., 2021; Zhou et al., 2022; Cheng et al., 2021). Adipose-derived mesenchymal stem cell exosomes (ADSC-Exo) inhibit ferroptosis in neurons by targeting CHAC1 with miR-760-3p, thereby reducing cerebral ischemia/reperfusion injury (Wang Y. et al., 2023). ADSC-Exo modified with PD-L1 and HGF target SDF-1α^+^ expression, creating an anti-inflammatory microenvironment in ischemic brain regions (Lin et al., 2024). A recent study showed that human umbilical cord mesenchymal stem cell-derived exosomes loaded with superparamagnetic iron oxide nanoparticles deliver miR-1228-5p to protect against oxidative damage and improve cognitive dysfunction after cerebral ischemia (Hu et al., 2024). Dental pulp stem cell-derived exosomes (DPSC-Exos) significantly inhibit neuroinflammation caused by I/R, reduce neuronal apoptosis through miR-877-3p, and alleviate brain edema, infarction, and neurological damage in I/R mice (Li S. et al., 2021; Miao et al., 2024). Neural stem cell-derived exosomes (NSC-Exo) improve brain tissue damage such as cerebral infarction, neuronal death, and glial scar formation, promoting motor function recovery. Their neuroprotective effects are associated with inducing M2 polarization of microglia and regulating the secretion of microglia-related inflammatory molecules, thereby alleviating inflammation (Zhang R. et al., 2023; Zhao X. et al., 2023). Astrocyte-derived exosomes (ATC-Exo) alleviate cerebral I/R injury by transporting miR-34c, which targets TLR7 and downregulates the NF-κB/MAPK signaling pathway (Wu et al., 2020b). Brain microvascular endothelial cell-derived exosomes (EC-Exos) play a crucial role in cell communication by promoting neuronal growth, migration, and invasion, directly protecting neurons from I/R injury (Xiao et al., 2017). Further studies show that EC-Exos significantly reduce Bcl2 and Caspase-3 expression while upregulating Bax in MCAO/R mouse brain tissue, weakening ischemia-induced neuronal apoptosis and benefiting ischemic stroke treatment (Sun et al., 2022). The latest research indicates that exercise-induced skeletal muscle-derived exosomes mitigate cerebral ischemic injury by regulating the miR-484/ACSL4 axis (Huang et al., 2024). Panax notoginseng-derived exosomes (PDN) can enter the brain without modification, improve cerebral infarction volume, enhance behavioral outcomes, and maintain BBB integrity. Similar to BMSC-derived exosomes, PDN also reduces cerebral I/R injury by shifting microglial phenotype from pro-inflammatory M1 to anti-inflammatory M2 (Li et al., 2023e). Exosomes from various sources have shown broad therapeutic potential in treating cerebral I/R injury. As a promising drug delivery vehicle, they warrant further investigation for their significant clinical implications.

**TABLE 2:** Exosome-based therapy for cerebral ischemia/reperfusion (I/R) injury.

Exosome source	Function	References
Circulating Plasma Exosomes (Pla-Exo)	Inhibit mitochondrial-mediated neuronal apoptosis and alleviate BBB damage via exosomal HSP70 mediated suppression of ROS	Jiang et al. (2020), Kang et al. (2019)
Bone Marrow Mesenchymal Stem Cell-Derived Exosomes (BMSC-Exos)	Restore lysosomal function via mTOR/TFEB	Liu et al. (2024a)
	Inhibit cell apoptosis and autophagy via targeting DAPK2/Akt with miR-133a	Yang et al. (2023b)
	Upregulate CXCR4, to regulate cell survival, promote angiogenesis and protect neurons	Li et al. (2020b)
	Promote M1 to M2 phenotype transition in microglia, alleviate neuronal pyroptosis	Liu et al. (2021), Zhou et al. (2022)
	Inhibit microglia apoptosis via miR-26a-5p mediated suppression of CDK6	Cheng et al. (2021)
Adipose-derived mesenchymal stem cell exosomes (ADSC-Exo)	Inhibit ferroptosis in neurons by targeting CHAC1 with miR-760-3p	Wang et al. (2023c)
Human umbilical cord mesenchymal stem cell-derived exosomes (hUCMSC -Exo)	Improve mitochondrial function and mitigate oxidative damage via activating TRAF6- NOX1 pathway with miR-1228-5p	Hu et al. (2024)
Dental Pulp Stem Cell-Derived Exosomes (DPSC-Exos)	Inhibit neuroinflammation and reduce neuronal apoptosis through miR-877-3p	Li et al. (2021c), Miao et al. (2024)
Neural Stem Cell (NSC)-Derived Exosomes	Reduce inflammation, oxidative stress, and promote NSCs differentiation	Zhang et al. (2023b), Zhao et al. (2023b)
Astrocyte-Derived Exosomes (ATC-Exo)	Protect neurons by downregulate NF-κB/MAPK pathway via targeting TLR7 with miR-34c	Wu et al. (2020b)
Brain Microvascular Endothelial Cell-Derived Exosomes (EC-Exos)	Promote synaptic remodeling and inhibit apoptosis from protecting neurons	Xiao et al. (2017), Sun et al. (2022)
Panax Notoginseng Derived-Exosomes (PDN)	Promote M1 to M2 phenotype transition in microglia via activating pI3k/Akt pathway	Li et al. (2023e)

### 3.5 Noncoding RNA therapeutics

miRNA, lncRNA, and circRNA play crucial regulatory roles in cerebral I/R injury and are considered vital biomarkers and therapeutic targets for the early diagnosis and prognosis assessment of stroke. These noncoding RNAs orchestrate complex pathological cascades through interactions with mRNAs, proteins, and signaling pathways, influencing neuroinflammation, oxidative stress, and programmed cell death. Exosomes, natural nanovesicles carrying functional miRNAs, have shown neuroprotective effects in preclinical I/R models by delivering miRNAs like miR-124 and miR-138-5p to neurons and glial cells, modulating apoptosis and mitochondrial function (Wang Y. et al., 2023; Hu et al., 2024; Miao et al., 2024; Wu et al., 2020a; Huang et al., 2024; Chen and Chopp, 2018). However, the clinical translation of noncoding RNA therapies faces challenges including rapid enzymatic degradation, poor BBB penetration, off-target effects, and immunogenicity. To overcome these limitations, innovative delivery systems are being engineered to enhance RNA stability and targeting specificity. For example, Yang et al. designed metal-organic framework nanoparticles (Ca-MOF@miR-124) to efficiently deliver miR-124 into neural stem cells (NSCs), enhancing their differentiation into functional neurons and reducing infarct volume in MCAO mice (Yang H. et al., 2022). Another study utilized microglia membrane-coated vesicles loaded with miR-138-5p (mMVs@miR-138-5p), exploiting the innate inflammatory chemotaxis of microglia to deliver the miRNA to ischemic neurons. This approach improved mitochondrial dynamics via the DNMT3A/Rhebl1 axis, attenuating neuronal death in rat I/R models (Zhu et al., 2024). For circRNA-targeted therapy, circular oxoglutarate dehydrogenase (circOGDH), a neuron-derived circRNA overexpressed in the ischemic penumbra of MCAO mice and human plasma exosomes, was silenced using siRNA-loaded PLGA-PAMAM nanoparticles (Liu Y. et al., 2022; Liu Y. et al., 2024). Systemic administration of these nanoparticles reduced neuronal apoptosis and improved functional recovery in preclinical models.

Clinically, miRNA-based therapies have advanced significantly, with the FDA and European Medicines Agency approving several RNA therapeutics, including anti-miR-92a (for vascular repair) and miR-34 mimics (for cancer), paving the way for stroke applications (Winkle et al., 2021; Liang et al., 2024). In contrast, lncRNA and circRNA therapies remain in early development, though emerging technologies show promise. For instance, CRISPR/Cas9-mediated silencing of MALAT1, a pro-inflammatory lncRNA, reduced neurovascular damage in murine I/R models (Khoshnam et al., 2024; Zhao N. et al., 2023). Similarly, nanoparticle-mediated delivery of XIST siRNA suppressed endothelial pyroptosis by targeting NLRP3 inflammasomes (Zhang et al., 2021). The convergence of gene-editing tools (e.g., CRISPR/dCas9) and advanced nanocarriers (e.g., lipid nanoparticles, exosome mimics) may unlock the therapeutic potential of lncRNAs and circRNAs, enabling precise modulation of neuroinflammatory pathways, BBB integrity, and neuronal survival. In summary, while miRNA therapies lead clinical translation efforts, lncRNA and circRNA-targeted strategies are poised to emerge as next-generation interventions. By integrating optimized delivery platforms with mechanistic insights into ncRNA networks, researchers aim to transform the treatment landscape for cerebral I/R injury, addressing unmet needs in stroke recovery and neuroprotection.

### 3.6 Combined intervention therapy

Emerging evidence highlights the critical role of multi-mechanistic interventions in addressing the spatiotemporal complexity of cerebral I/R injury. Recent advances in nanomaterial engineering and drug synergy strategies now enable precise targeting of cascading pathological events—from acute oxidative bursts to delayed neuroinflammation and apoptosis—while preserving neurovascular unit integrity. This paradigm shift underscores the therapeutic imperative to integrate phase-specific pharmacological actions with biomimetic drug delivery, harmonizing acute cytoprotection with long-term tissue remodeling.

Co-administration of Edaravone dexborneol enhances free radical scavenging, improving 3-month functional independence rates (modified Rankin Scale score ≤2) by 2.3-fold compared to tPA alone (Dang et al., 2024). Concurrently, the combined application of mild hypothermia (MH) and edaravone (EDA) exerts synergistic neuroprotection against cerebral ischemia-reperfusion injury through Nrf2 pathway activation, effectively reversing neurological deficits and oxidative stress in mice (Yu et al., 2020). Advanced drug delivery systems, such as pH/ROS-responsive nanocarriers, would achieve lesion-specific drug release within ischemic tissue while neutralising ROS in real time (Chen W. et al., 2021). Nanomaterial-engineered systems - including NBP-CeO2NPs, HD@iNanoAOX, and AgNPs delivering agomelatine - demonstrate enhanced neuroprotection by integrating BBB stabilization, hypoxia-responsive drug release, and inhibition of oxidative stress, effectively reducing infarction volume and neuronal apoptosis in preclinical cerebral ischemic models (Li X. et al., 2022; Mei et al., 2025; Gelen et al., 2024). The bioinspired PB-006@MSC nanoplatform integrates a Prussian blue scavenger, neuroprotectant ZL006, and stem cell membrane targeting to achieve precise ischemic penumbra delivery (Zhang Q. et al., 2024). Its multidimensional mechanisms encompass ROS clearance, PSD95 suppression, and inflammatory regulation, culminating in significantly reduced cerebral infarction and improved neurological recovery. Combinatorial therapies utilizing exosomes and anti-apoptotic RNA agents effectively attenuate neuroinflammation, reduce neuronal apoptosis in the ischemic penumbra, improve cognitive function, and restore blood-brain barrier integrity (Yang X. et al., 2023; Lin et al., 2024; Hu et al., 2024). Additionally, the combination of BBB stabilizers and neuroprotective agents also demonstrates promising efficacy in the treatment of cerebral ischemia-reperfusion injury (Ugale et al., 2025). A recent study developed SRPNNPs, a polysorbate 80-modified carrier-free nanoformulation co-assembling ginsenoside Rb1, 3-n-butylphthalide, and probucol, which targets cerebral ischemia-reperfusion injury through apolipoprotein-mediated BBB penetration, neuronal and microglial internalization, and synergistic antioxidant/anti-inflammatory/neuroprotective effects (Guo et al., 2025). Collectively, these stratified therapeutic regimens demonstrate the critical importance of phase-specific multi-target interventions in cerebral I/R injury management, while underscoring the need for further translational research to optimize their synergistic mechanisms and temporal coordination.

## 4 Perspectives and prospects

With the advancement of medical technology, significant progress has been made in the treatment of cerebral I/R injury. Traditional drug therapy, antioxidants, neuroprotective agents, exosomes, and noncoding RNA therapeutics have each demonstrated their unique advantages and translational challenges. Drug therapy remains one of the primary approaches for treating cerebral I/R injury. Traditional drugs derived from natural plant compounds or compound combinations can alleviate cerebral I/R injury through multiple mechanisms, including antioxidation, anti-neuroinflammation, and regulation of autophagy, yet their multi-target nature complicates mechanistic validation in human trials. Clinical research data have shown the effectiveness of antioxidants and neuroprotective agents, while nano-drug delivery systems enhance their bioavailability through real-time oxidative stress neutralization and lesion-specific release, though scale-up manufacturing and batch-to-batch consistency remain hurdles for clinical adoption.

Exosomes derived from stem cells utilize miRNAs to promote neural repair, but challenges in scalable GMP-compliant production require microfluidic standardization and improved biodistribution tracking techniques to meet regulatory requirements. Noncoding RNA therapeutics targeting key pathways show promise, yet require AI-guided pharmacokinetic modeling to optimize dosing sequences and mitigate hemorrhagic transformation risks, particularly given inter-patient variability in RNA metabolism. The current limitations stem from biological heterogeneity and low blood-brain barrier penetration efficiency. These issues are now expected to be addressed by 3D bioprinted neurovascular units for blood-brain barrier permeability screening and CRISPR-engineered exosome-mimicking vesicles with hypoxia-responsive surface ligands for ischemia targeting. To bridge the preclinical-clinical gap, future strategies must integrate nanomaterials, temporal therapeutic algorithms, and multi-omics profiling to develop closed-loop systems capable of auto-calibrating drug release based on real-time biomarker feedback, validated through multicenter trials with phenotypically stratified cohorts.
